# Supplementary material for: Multimodal fusion of structural and functional brain imaging in depression using linked independent component analysis
Source: Hum Brain Mapp. 2019 Oct 1;41(1):241–55. doi: 10.1002/hbm.24802 (PMC7267936; doi:10.1002/hbm.24802)
Supplement: Supplementary file 1 — Appendix S1: Supporting Information [file HBM-41-241-s001.docx]

**Multimodal fusion of structural and functional brain imaging in depression using linked independent component analysis**

***Supplemental Information***

**Supplemental Methods**

*Accounting for other confounds*

The supplemental analyses to account for various confounders described below were performed based on using the same feature set as the main analyses. Specifically, this was the LICA decomposition of 40 ICs with age and sex residualized out from all the subject weights of all the ICs, and additionally phase encoding from IC4.

Imbalanced group sizes: Here we randomly selected two groups of equal size from the case and control samples 100 times and performed the case-control classification (i.e. 10-fold cross validation with 100 repetitions) for each of these randomly selected groups.

Depression severity: We conducted case-control classification where we excluded

cases with minimal depression severity based on BDI sum score criteria (>= 14; n = 110). We also exclude controls with moderate depression severity based on BDI sum score criteria (>= 20; n = 1, with an additional control with no symptom data).

SSRI medication use: Here, we regressed out SSRI medication use from all the subject weights of the 40 ICs for case-control classification, and for predicting symptom loads of depression and anxiety.

**Supplemental Results**

Machine learning results where age, sex and phase encoding direction have been regressed out from the subject weights of all the IC features.

*Case-control classification*

AUC = 0.60937, *p* = 0.1357, accuracy = 0.5351, sensitivity = 0.6333, specificity = 0.3092.

*Predicting symptom loads of depression*

RMSE = 11.077, *p* = 0.9759, MAE = 8.801, R^2^ = -0.4142, spearman’s rho = -0.0994

*Predicting symptom loads of anxiety*

RMSE = 8.239, *p* = 0.9104, MAE = 6.334, R^2^ = -0.445, spearman’s rho = -0.1022.

Machine learning results where phase age and sex have been regressed out from all the IC features, and phase encoding direction from IC4 at the higher decomposition (80, with a total of 67 components).

*Case-control classification*

AUC = 0.6046, *p* = 0.1828, accuracy = 0.5813, sensitivity = 0.6683, specificity = 0.37645

*Predicting symptom loads of depression*

RMSE = 11.786, *p* = 0.9984, MAE = 9.180, R^2^ = -0.6126, spearman’s rho = 0.009

*Predicting symptom loads of anxiety*

RMSE = 9.235, *p* = 0.9962, MAE = 7.0402, R^2^ = -0.8693, spearman’s rho = -0.1019

Machine learning results for predicting age when regressing out phase encoding direction from the subject weights of all the ICs:

RMSE = 7.246, *p* < 0.0001, MAE = 5.988, R^2^ = 0.671, r = 0.838

Machine learning results for predicting age when regressing out phase encoding direction from IC4 in the higher model order decomposition (80, with a total of 67 components):

RMSE = 7.331, *p* < 0.0001, MAE = 5.942, R^2^ = 0.662, r = 0.838

Controlling for unequal sample size, depression severity, and SSRI status

Machine learning results for classifying cases and controls, with random undersampling repeated 100 times

AUC = 0.632, accuracy = 0.530, sensitivity = 0.413, specificity = 0.647

Machine learning results for classifying cases and controls, excluding the control with moderate depression and all cases with minimal depression

AUC = 0.636, accuracy = 0.568, sensitivity = 0.443, specificity = 0.685

Machine learning analyses regressing out SSRI status from the subject weights of all the ICs

Classification results:

AUC = 0.598, accuracy = 0.584, sensitivity = 0.664, specificity = 0.400

Depression symptom load:

RMSE = 10.83, MAE = 8.59, R^2^ = -0.357, spearman’s rho = -0.023

Anxiety symptom load:

RMSE = 8.22, MAE = 6.30, R^2^ = -0.435, spearman’s rho = -0.098

**Supplemental Tables**

**Table S1**. Interaction effects of group, symptom loads of depression (BDI) and anxiety (BAI) with age and sex on ICs. The p-values are FDR-corrected

| **IC** | **Group x age (*t, p*)** | **Group x sex (*t, p*)** | **BDI x age**  **(*t, p*)** | **BDI x sex**  **(*t, p*)** | **BAI x age**  **(*t, p*)** | **BAI x sex**  **(*t, p*)** |
| --- | --- | --- | --- | --- | --- | --- |
| IC0 | -0.14 (0.947) | -0.22 (0.912) | -1.06 (0.899) | -1.10 (0.641) | 0.11 (0.976) | -0.82 (0.958) |
| IC1 | 0.31 (0.922) | 0.50 (0.823) | -0.34 (0.899) | 1.14 (0.641) | -0.55 (0.895) | 0.66 (0.958) |
| IC2 | 1.31 (0.813) | -0.17 (0.912) | 0.72 (0.899) | -1.11 (0.641) | 1.24 (0.895) | 0.30 (0.958) |
| IC3 | -0.40 (0.865) | 1.13 (0.823) | 0.36 (0.899) | 0.14 (0.982) | -1.24 (0.895) | -0.71 (0.958) |
| IC4 | 0.85 (0.813) | 1.05 (0.823) | 0.40 (0.899) | 0.61 (0.982) | 0.70 (0.895) | -0.15 (0.958) |
| IC5 | -0.65 (0.827) | -1.71 (0.587) | 0.44 (0.899) | -1.2 (0.641) | 1.08 (0.895) | 1.20 (0.788) |
| IC6 | 0.43 (0.865) | 0.56 (0.823) | 0.18 (0.956) | 0.58 (0.982) | -0.07 (0.976) | 0.27 (0.958) |
| IC7 | -0.79 (0.813) | -2.21 (0.379) | -0.47 (0.899) | -2.19 (0.479) | -0.90 (0.895) | -1.70 (0.609) |
| IC8 | 2.52 (0.167) | 2.20 (0.379) | 1.05 (0.899) | 0.24 (0.982) | 0.28 (0.976) | 0.20 (0.958) |
| IC9 | -0.79 (0.813) | 0.57 (0.823) | -0.49 (0.899) | 0.49 (0.982) | -0.76 (0.895) | -0.32 (0.958) |
| IC10 | 0.73 (0.816) | 0.67 (0.823) | 0.015 (0.988) | -0.16 (0.982) | 0.43 (0.895) | 0.34 (0.958) |
| IC11 | 0.82 (0.813) | 0.65 (0.823) | 0.29 (0.899) | 0.05 (0.982) | -0.03 (0.976) | -0.07 (0.958) |
| IC12 | -0.98 (0.813) | 1.42 (0.786) | -1.73 (0.703) | -1.12 (0.641) | -1.57 (0.895) | -1.74 (0.609) |
| IC13 | 0.76 (0.813) | 0.45 (0.823) | 0.84 (0.899) | 0.80 (0.942) | 1.48 (0.895) | 0.69 (0.958) |
| IC14 | 0.17 (0.947) | 0.46 (0.823) | 0.81 (0.899) | 0.26 (0.982) | 0.52 (0.895) | 0.24 (0.958) |
| IC15 | -1.88 (0.487) | 1.32 (0.823) | -0.57 (0.899) | 0.10 (0.982) | 0.06 (0.976) | -0.10 (0.958) |
| IC16 | 1.05 (0.813) | -0.17 (0.912) | 2.08 (0.703) | 0.10 (0.982) | 1.21 (0.895) | -0.42 (0.958) |
| IC17 | 2.08 (0.388) | 0.192 (0.912) | 0.06 (0.988) | -2.09 (0.479) | -0.64 (0.895) | -2.19 (0.609) |
| IC18 | 0.05 (0.962) | -0.08 (0.962) | 0.03 (0.988) | -1.39 (0.641) | -0.47 (0.895) | -1.13 (0.803) |
| IC19 | -2.53 (0.167) | 0.51 (0.823) | -0.39 (0.899) | -0.13 (0.982) | -0.71 (0.895) | -0.20 (0.958) |
| IC20 | 0.11 (0.947) | 0.17 (0.912) | 0.42 (0.899) | 0.24 (0.982) | 0.85 (0.895) | 0.48 (0.958) |
| IC21 | 2.53 (0.167) | 0.75 (0.823) | 1.63 (0.703) | -0.37 (0.982) | 0.45 (0.895) | -0.78 (0.958) |
| IC22 | 1.41 (0.813) | 1.72 (0.587) | 1.82 (0.703) | 1.58 (0.641) | 1.73 (0.895) | 0.60 (0.958) |
| IC23 | -1.32 (0.813) | -0.05 (0.963) | -1.63 (0.703) | -1.16 (0.641) | -2.20 (0.895) | -1.34 (0.729) |
| IC24 | 0.60 (0.827) | 2.59 (0.379) | 1.72 (0.703) | -0.20 (0.982) | 0.37 (0.921) | -0.21 (0.958) |
| IC25 | -1.20 (0.813) | 0.87 (0.823) | 0.33 (0.899) | 0.30 (0.982) | 0.65 (0.895) | 1.42 (0.729) |
| IC26 | -0.81 (0.813) | 0.76 (0.823) | 0.44 (0.899) | 1.99 (0.479) | -0.43 (0.895) | 0.56 (0.958) |
| IC27 | 1.06 (0.813) | -0.57 (0.823) | 0.47 (0.899) | -1.50 (0.641) | -0.64 (0.895) | -1.81 (0.609) |
| IC28 | -0.79 (0.813) | 0.90 (0.823) | 1.35 (0.871) | 1.68 (0.641) | 1.30 (0.895) | 1.95 (0.609) |
| IC29 | 0.27 (0.924) | 0.90 (0.823) | -0.40 (0.899) | -0.06 (0.982) | -0.81 (0.895) | 0.10 (0.958) |
| IC30 | 0.61 (0.827) | -0.80 (0.823) | -1.30 (0.871) | -1.39 (0.641) | -1.33 (0.895) | -2.17 (0.609) |
| IC31 | 1.15 (0.813) | 0.56 (0.823) | 0.60 (0.899) | 0.33 (0.982) | 0.07 (0.976) | -1.19 (0.788) |
| IC32 | -0.46 (0.865) | -0.49 (0.823) | 0.32 (0.899) | 0.13 (0.982) | -0.11 (0.976) | -1.44 (0.729) |
| IC33 | 0.10 (0.947) | 1.26 (0.823) | -0.27 (0.899) | 0.001 (0.999) | 0.03 (0.976) | -0.43 (0.958) |
| IC34 | 0.40 (0.865) | 0.41 (0.831) | 0.62 (0.899) | 1.14 (0.641) | 0.78 (0.895) | 0.48 (0.958) |
| IC35 | 0.59 (0.827) | 1.43 (0.786) | 0.49 (0.899) | 0.16 (0.982) | 0.16 (0.976) | -0.30 (0.958) |
| IC36 | 0.94 (0.813) | 1.80 (0.587) | 1.32 (0.871) | 2.14 (0.479) | 0.86 (0.895) | 0.89 (0.958) |
| IC37 | 1.14 (0.813) | -0.69 (0.823) | 0.10 (0.988) | -1.29 (0.641) | -0.51 (0.895) | -1.39 (0.729) |
| IC38 | -0.18 (0.947) | 0.44 (0.823) | -0.35 (0.899) | 0.15 (0.982) | -0.49 (0.895) | -0.83 (0.958) |
| IC39 | -0.46 (0.865) | -0.46 (0.823) | -1.06 (0.899) | 0.17 (0.982) | -0.47 (0.895) | 0.05 (0.958) |

**Table S2**. Main effects of age, sex, Group, symptom loads of depression (BDI), and anxiety (BAI) on ICs from the higher model order decomposition. The p-values are FDR-corrected

| **IC** | **Age (*t, p*)** | **Sex (*t, p*)** | **Group (*t, p*)** | **BDI (*t, p*)** | **BAI (*t, p*)** |
| --- | --- | --- | --- | --- | --- |
| IC0 | -7.54 (<0.001) | 8.92 (<0.001) | 0.47 (0.905) | -0.15 (0.962) | 0.56 (0.949) |
| IC1 | 7.29 (<0.001) | 0.78 (0.649) | 0.96 (0.905) | 1.24 (0.946) | -0.19 (0.949) |
| IC2 | -12.44 (<0.001) | -10.75 (<0.001) | 0.73 (0.905) | 0.09 (0.962) | -0.50 (0.949) |
| IC3 | 0.24 (0.889) | -4.09 (0.001) | 0.61 (0.905) | 0.65 (0.961) | 0.75 (0.949) |
| IC4 | -6.55 (<0.001) | 1.89 (0.252) | 1.30 (0.905) | 0.36 (0.962) | 0.40 (0.949) |
| IC8 | -4.81 (<0.001) | 2.23 (0.139) | 0.87 (0.905) | -1.26 (0.946) | -2.29 (0.767) |
| IC15 | -6.12 (<0.001) | 3.17 (0.018) | -1.19 (0.905) | -1.16 (0.946) | -0.89 (0.949) |
| IC16 | -1.12 (0.568) | 2.36 (0.108) | -0.63 (0.905) | 0.11 (0.962) | 0.19 (0.949) |
| IC17 | -1.03 (0.568) | -2.55 (0.069) | 0.63 (0.905) | 0.38 (0.962) | 1.04 (0.949) |
| IC20 | 1.57 (0.341) | -0.85 (0.649) | -0.15 (0.964) | 0.59 (0.961) | 0.39 (0.949) |
| IC22 | 1.19 (0.551) | -0.22 (0.906) | -1.13 (0.905) | -0.61 (0.961) | -0.55 (0.949) |
| IC23 | -0.98 (0.572) | -0.32 (0.865) | -0.29 (0.964) | 0.56 (0.962) | 0.17 (0.949) |
| IC25 | -3.16 (0.012) | 1.05 (0.55) | -0.81 (0.905) | -0.47 (0.962) | -0.36 (0.949) |
| IC26 | -2.25 (0.106) | 1.68 (0.289) | 2.35 (0.905) | -0.68 (0.961) | 0.55 (0.949) |
| IC27 | -1.05 (0.568) | 1.69 (0.289) | -0.18 (0.964) | 0.70 (0.961) | 0.56 (0.949) |
| IC28 | 6.01 (<0.001) | 0.52 (0.807) | 0.72 (0.905) | 1.31 (0.946) | 0.23 (0.949) |
| IC29 | 0.31 (0.86) | 0.16 (0.91) | -1.09 (0.905) | -0.85 (0.961) | -0.71 (0.949) |
| IC30 | -0.80 (0.607) | 3.14 (0.018) | -0.52 (0.905) | -1.49 (0.946) | -1.29 (0.949) |
| IC31 | 1.90 (0.195) | -3.93 (0.001) | 0.67 (0.905) | -0.08 (0.962) | -0.22 (0.949) |
| IC32 | -0.45 (0.781) | -0.42 (0.833) | 1.33 (0.905) | 0.18 (0.962) | -0.28 (0.949) |
| IC33 | 2.57 (0.052) | -1.54 (0.322) | 0.42 (0.905) | -1.08 (0.946) | -1.40 (0.949) |
| IC34 | -2.96 (0.02) | 5.29 (<0.001) | 1.44 (0.905) | -0.31 (0.962) | -0.10 (0.949) |
| IC35 | -0.91 (0.593) | 3.09 (0.019) | 1.50 (0.905) | 1.31 (0.946) | 0.26 (0.949) |
| IC36 | 0.83 (0.593) | -2.73 (0.051) | 1.65 (0.905) | 1.33 (0.946) | 1.05 (0.949) |
| IC37 | 0.15 (0.923) | 0.82 (0.649) | -0.72 (0.905) | -1.65 (0.946) | -1.52 (0.949) |
| IC38 | -0.50 (0.768) | 1.91 (0.252) | 0.75 (0.905) | 0.12 (0.962) | -0.24 (0.949) |
| IC39 | -1.09 (0.568) | -0.15 (0.91) | 0.23 (0.964) | 0.01 (0.994) | -0.10 (0.949) |
| IC40 | -4.80 (<0.001) | -0.38 (0.843) | -0.42 (0.905) | -0.27 (0.962) | 0.03 (0.988) |
| IC41 | 1.18 (0.551) | 1.84 (0.259) | 1.018 (0.905) | 0.71 (0.961) | 0.78 (0.949) |
| IC42 | 0.86 (0.593) | 1.09 (0.55) | -1.37 (0.905) | -1.05 (0.946) | -0.60 (0.949) |
| IC43 | 0.58 (0.727) | 1.55 (0.322) | 0.66 (0.905) | 2.18 (0.946) | 1.40 (0.949) |
| IC44 | -0.74 (0.632) | -1.26 (0.457) | 0.37 (0.92) | -0.36 (0.962) | -0.56 (0.949) |
| IC45 | -0.97 (0.572) | 0.71 (0.679) | -0.20 (0.964) | -1.06 (0.946) | -1.60 (0.949) |
| IC46 | -1.50 (0.362) | 0.22 (0.906) | -0.48 (0.905) | -0.54 (0.962) | -0.66 (0.949) |
| IC47 | 0.60 (0.718) | -1.77 (0.259) | 0.59 (0.905) | 0.21 (0.962) | 0.46 (0.949) |
| IC48 | -0.41 (0.787) | 1.46 (0.362) | -0.06 (0.987) | 0.23 (0.962) | 0.37 (0.949) |
| IC49 | 0.86 (0.593) | 0.59 (0.778) | -0.40 (0.905) | 0.71 (0.961) | -0.20 (0.949) |
| IC50 | -8.93 (<0.001) | -0.34 (0.864) | -0.02 (0.987) | 1.23 (0.946) | 2.13 (0.77) |
| IC51 | -2.78 (0.033) | 0.79 (0.649) | 0.52 (0.905) | 0.62 (0.961) | -0.15 (0.949) |
| IC52 | -1.79 (0.237) | 1.40 (0.391) | -0.26 (0.964) | -0.30 (0.962) | -0.39 (0.949) |
| IC53 | 1.25 (0.531) | -0.95 (0.597) | -0.04 (0.987) | 0.19 (0.962) | 0.71 (0.949) |
| IC54 | 1.95 (0.183) | -2.15 (0.155) | 0.44 (0.905) | -0.96 (0.961) | -0.43 (0.949) |
| IC55 | -0.42 (0.787) | 1.08 (0.55) | -0.02 (0.987) | 1.71 (0.946) | 0.94 (0.949) |
| IC56 | -2.10 (0.144) | 0.15 (0.91) | 1.82 (0.905) | 0.59 (0.961) | 0.57 (0.949) |
| IC57 | -0.04 (0.967) | 1.34 (0.418) | 1.49 (0.905) | 0.64 (0.961) | 0.33 (0.949) |
| IC58 | -0.76 (0.622) | 1.57 (0.322) | -0.42 (0.905) | -0.35 (0.962) | -0.74 (0.949) |
| IC59 | -1.64 (0.311) | 1.03 (0.55) | -0.89 (0.905) | -0.59 (0.961) | -0.83 (0.949) |
| IC60 | 0.84 (0.593) | -1.15 (0.525) | -0.13 (0.972) | 1.55 (0.946) | 0.92 (0.949) |
| IC61 | 0.52 (0.765) | -1.03 (0.55) | 1.10 (0.905) | 0.59 (0.961) | 0.24 (0.949) |
| IC62 | 0.47 (0.78) | -2.69 (0.051) | 1.45 (0.905) | -0.94 (0.961) | -0.89 (0.949) |
| IC63 | -0.89 (0.593) | -0.83 (0.649) | 0.59 (0.905) | 1.20 (0.946) | -0.11 (0.949) |
| IC64 | -1.45 (0.386) | 1.64 (0.299) | 0.25 (0.964) | 1.11 (0.946) | 0.92 (0.949) |
| IC65 | 0.05 (0.967) | 1.78 (0.259) | 0.23 (0.964) | -0.79 (0.961) | -1.72 (0.949) |
| IC66 | 1.06 (0.568) | -1.80 (0.259) | -0.17 (0.964) | -0.18 (0.962) | -0.50 (0.949) |
| IC67 | 2.03 (0.161) | -0.05 (0.971) | -1.11 (0.905) | -0.38 (0.962) | -0.41 (0.949) |
| IC68 | -0.14 (0.923) | 0.26 (0.904) | 0.53 (0.905) | 0.05 (0.977) | -0.02 (0.988) |
| IC69 | 0.23 (0.889) | 0.79 (0.649) | 1.13 (0.905) | 0.27 (0.962) | 0.14 (0.949) |
| IC70 | 0.99 (0.572) | 0.45 (0.825) | -1.13 (0.905) | -0.40 (0.962) | -0.44 (0.949) |
| IC71 | 1.03 (0.568) | -0.16 (0.91) | -0.52 (0.905) | -0.77 (0.961) | 0.23 (0.949) |
| IC72 | -1.04 (0.568) | 0.71 (0.679) | 0.05 (0.987) | 0.66 (0.961) | 0.79 (0.949) |
| IC73 | -2.34 (0.089) | -1.25 (0.457) | 0.81 (0.905) | 0.12 (0.962) | 0.88 (0.949) |
| IC74 | 1.52 (0.36) | -0.55 (0.796) | -0.75 (0.905) | -0.26 (0.962) | 0.36 (0.949) |
| IC75 | -2.57 (0.052) | 0.50 (0.808) | -0.98 (0.905) | 0.12 (0.962) | -0.31 (0.949) |
| IC76 | -0.22 (0.889) | 0.94 (0.597) | -0.70 (0.905) | -1.49 (0.946) | -0.95 (0.949) |
| IC77 | 0.90 (0.593) | -0.40 (0.843) | 0.82 (0.905) | -1.43 (0.946) | -0.34 (0.949) |
| IC78 | -0.65 (0.694) | -0.49 (0.808) | 0.78 (0.905) | 1.13 (0.946) | 2.35 (0.767) |
| IC79 | -0.13 (0.923) | -0.02 (0.985) | 0.82 (0.905) | 1.83 (0.946) | 0.56 (0.949) |

**Table S3**. Interaction effects of group, symptom loads of depression (BDI) and anxiety (BAI) with age and sex on ICs from the higher model order decomposition. The p-values are FDR-corrected.

| **IC** | **Group x age (*t, p*)** | **Group x sex (*t, p*)** | **BDI x age**  **(*t, p*)** | **BDI x sex**  **(*t, p*)** | **BAI x age**  **(*t, p*)** | **BAI x sex**  **(*t, p*)** |
| --- | --- | --- | --- | --- | --- | --- |
| IC0 | 1.20 (0.664) | 1.37 (0.774) | 0.57 (0.898) | 1.46 (0.786) | 0.02 (0.988) | 1.10 (0.85) |
| IC1 | -0.10 (0.951) | -0.30 (0.952) | -1.04 (0.863) | -1.12 (0.786) | 0.11 (0.986) | -0.78 (0.965) |
| IC2 | 1.28 (0.664) | -0.66 (0.854) | 0.3 (0.902) | -1.37 (0.786) | 0.85 (0.914) | -0.21 (0.965) |
| IC3 | -0.80 (0.791) | 0.57 (0.854) | 0.06 (0.978) | -0.18 (0.975) | -1.72 (0.754) | -1.09 (0.85) |
| IC4 | 1.38 (0.664) | 0.85 (0.854) | 0.83 (0.884) | -0.01 (0.993) | 0.97 (0.914) | -0.85 (0.965) |
| IC8 | 0.43 (0.914) | 0.31 (0.952) | -0.40 (0.902) | 0.70 (0.799) | -0.25 (0.986) | 0.29 (0.965) |
| IC15 | -0.76 (0.791) | -1.70 (0.665) | 0.57 (0.898) | -1.11 (0.786) | 1.04 (0.914) | 1.18 (0.85) |
| IC16 | -0.43 (0.914) | -0.28 (0.952) | 0.43 (0.902) | 0.12 (0.993) | 0.78 (0.914) | 0.36 (0.965) |
| IC17 | 0.14 (0.951) | -0.74 (0.854) | 1.18 (0.863) | -0.50 (0.799) | 0.18 (0.986) | -0.03 (0.986) |
| IC20 | 0.20 (0.951) | 0.97 (0.854) | 0.46 (0.902) | 0.49 (0.799) | -0.16 (0.986) | -0.57 (0.965) |
| IC22 | 1.40 (0.664) | 0.10 (0.961) | 0.94 (0.863) | 0.06 (0.993) | 0.71 (0.914) | 0.35 (0.965) |
| IC23 | 1.18 (0.664) | 0.61 (0.854) | 0.11 (0.955) | -0.07 (0.993) | 0.44 (0.94) | 0.12 (0.978) |
| IC25 | 0.12 (0.951) | -0.69 (0.854) | -0.50 (0.902) | 0.74 (0.799) | -0.54 (0.937) | -1.09 (0.85) |
| IC26 | -0.53 (0.911) | 0.88 (0.854) | 0.87 (0.884) | 0.78 (0.799) | 0.15 (0.986) | 0.58 (0.965) |
| IC27 | 1.41 (0.664) | -0.49 (0.854) | -0.02 (0.988) | -1.78 (0.786) | -0.74 (0.914) | -2.59 (0.559) |
| IC28 | 0.50 (0.911) | -0.91 (0.854) | -1.62 (0.794) | -1.49 (0.786) | -1.53 (0.846) | -2.16 (0.559) |
| IC29 | 1.49 (0.664) | -0.14 (0.961) | 1.48 (0.802) | -1.37 (0.786) | 0.38 (0.986) | -1.52 (0.85) |
| IC30 | 0.13 (0.951) | 1.66 (0.665) | 0.76 (0.896) | 0.78 (0.799) | 0.79 (0.914) | 1.20 (0.85) |
| IC31 | 1.35 (0.664) | 0.89 (0.854) | 2.18 (0.51) | 1.43 (0.786) | 1.98 (0.754) | 0.97 (0.908) |
| IC32 | 1.67 (0.664) | 0.71 (0.854) | 2.42 (0.51) | 1.08 (0.786) | 2.22 (0.754) | 0.33 (0.965) |
| IC33 | -0.81 (0.791) | 0.03 (0.974) | -1.23 (0.863) | -1.41 (0.786) | -1.92 (0.754) | -1.46 (0.85) |
| IC34 | -1.43 (0.664) | -0.11 (0.961) | -0.96 (0.863) | -1.87 (0.786) | -0.22 (0.986) | -0.13 (0.978) |
| IC35 | 1.28 (0.664) | -0.07 (0.961) | 2.32 (0.51) | -0.32 (0.884) | 1.77 (0.754) | 0.21 (0.965) |
| IC36 | 2.11 (0.608) | 2.46 (0.425) | 1.00 (0.863) | 0.10 (0.993) | 0.84 (0.914) | 0.40 (0.965) |
| IC37 | 0.49 (0.911) | -0.11 (0.961) | 0.56 (0.898) | -1.27 (0.786) | -0.24 (0.986) | -1.63 (0.85) |
| IC38 | -0.35 (0.919) | -1.14 (0.774) | -1.43 (0.802) | -1.86 (0.786) | -1.27 (0.914) | -2.15 (0.559) |
| IC39 | 0.70 (0.793) | 0.31 (0.952) | 0.71 (0.898) | 1.20 (0.786) | 1.06 (0.914) | 0.24 (0.965) |
| IC40 | 1.01 (0.781) | 0.83 (0.854) | 1.10 (0.863) | 1.12 (0.786) | 1.65 (0.754) | 1.37 (0.85) |
| IC41 | 1.43 (0.664) | 3.18 (0.111) | 1.13 (0.863) | 0.25 (0.926) | 0.49 (0.937) | -0.65 (0.965) |
| IC42 | -0.23 (0.951) | 0.51 (0.854) | 0.63 (0.898) | -0.44 (0.993) | 0.65 (0.914) | -0.28 (0.965) |
| IC43 | -1.52 (0.664) | -0.50 (0.854) | 0.95 (0.863) | 1.42 (0.786) | 0.74 (0.914) | 0.96 (0.908) |
| IC44 | 1.35 (0.664) | 0.94 (0.854) | 1.66 (0.794) | 0.96 (0.799) | -0.09 (0.986) | -0.37 (0.965) |
| IC45 | -0.74 (0.791) | 1.71 (0.665) | 0.54 (0.898) | 0.74 (0.799) | 0.13 (0.986) | 0.23 (0.965) |
| IC46 | -1.33 (0.664) | 0.66 (0.854) | -1.02 (0.863) | -0.41 (0.827) | -0.61 (0.914) | -0.32 (0.965) |
| IC47 | 2.56 (0.374) | 0.82 (0.854) | 2.27 (0.51) | -0.36 (0.865) | 1.31 (0.914) | -0.40 (0.965) |
| IC48 | -0.38 (0.919) | -0.25 (0.961) | 0.32 (0.902) | 1.41 (0.786) | -0.08 (0.986) | 0.60 (0.965) |
| IC49 | -0.21 (0.951) | 0.17 (0.961) | -1.77 (0.794) | -1.04 (0.799) | -1.96 (0.754) | -1.23 (0.85) |
| IC50 | 1.66 (0.664) | 0.51 (0.854) | -0.23 (0.902) | -0.51 (0.799) | -0.46 (0.94) | -1.26 (0.85) |
| IC51 | 0.36 (0.919) | -1.48 (0.774) | 0.23 (0.902) | -0.96 (0.799) | 0.06 (0.986) | -1.18 (0.85) |
| IC52 | 1.18 (0.664) | 0.55 (0.854) | 0.23 (0.902) | -0.50 (0.799) | 0.07 (0.986) | 0.02 (0.986) |
| IC53 | 0.76 (0.791) | 0.13 (0.961) | 0.69 (0.898) | 1.081 (0.786) | -0.26 (0.986) | -0.55 (0.965) |
| IC54 | -0.08 (0.953) | 0.29 (0.952) | -0.37 (0.902) | -0.69 (0.799) | -0.80 (0.914) | -1.00 (0.908) |
| IC55 | -0.01 (0.995) | -0.65 (0.854) | 1.42 (0.802) | -0.83 (0.799) | 1.20 (0.914) | 0.31 (0.965) |
| IC56 | -3.16 (0.118) | -0.61 (0.854) | -2.07 (0.535) | -0.92 (0.799) | -2.36 (0.754) | -2.14 (0.559) |
| IC57 | -1.26 (0.664) | 1.35 (0.774) | -0.59 (0.898) | 0.77 (0.799) | -0.51 (0.937) | 0.07 (0.986) |
| IC58 | -0.81 (0.791) | -0.80 (0.854) | -1.24 (0.863) | -1.44 (0.786) | -0.74 (0.914) | -1.27 (0.85) |
| IC59 | 0.91 (0.781) | -2.09 (0.425) | 1.57 (0.797) | -1.13 (0.786) | 0.93 (0.914) | -0.26 (0.965) |
| IC60 | 0.37 (0.919) | 2.18 (0.425) | 0.46 (0.902) | 0.65 (0.799) | 0.52 (0.937) | 1.27 (0.85) |
| IC61 | -0.89 (0.781) | 1.81 (0.665) | 0.36 (0.902) | -0.02 (0.993) | 0.03 (0.988) | 0.31 (0.965) |
| IC62 | 0.29 (0.921) | 0.11 (0.961) | 0.91 (0.873) | -0.43 (0.827) | 1.08 (0.914) | -0.19 (0.965) |
| IC63 | 0.91 (0.781) | 0.29 (0.952) | 0.28 (0.902) | -0.53 (0.799) | 0.61 (0.914) | -1.16 (0.85) |
| IC64 | -1.00 (0.781) | -2.27 (0.425) | -0.03 (0.988) | -1.09 (0.786) | 0.12 (0.986) | -0.76 (0.965) |
| IC65 | 0.32 (0.921) | 1.20 (0.774) | 0.55 (0.898) | 1.57 (0.786) | 0.29 (0.986) | 0.56 (0.965) |
| IC66 | 0.30 (0.921) | -0.77 (0.854) | -0.64 (0.898) | -2.01 (0.786) | -1.47 (0.875) | -1.42 (0.85) |
| IC67 | 2.16 (0.608) | 1.15 (0.774) | -1.28 (0.863) | -0.52 (0.799) | -1.29 (0.914) | 0.20 (0.965) |
| IC68 | -0.70 (0.793) | 0.08 (0.961) | 0.28 (0.902) | 0.04 (0.993) | 0.72 (0.914) | -0.29 (0.965) |
| IC69 | 1.04 (0.781) | 1.23 (0.774) | 0.37 (0.902) | -0.57 (0.799) | -0.35 (0.986) | -1.32 (0.85) |
| IC70 | -0.79 (0.791) | -1.18 (0.774) | -0.80 (0.894) | -0.66 (0.799) | -0.63 (0.914) | -0.25 (0.965) |
| IC71 | 0.17 (0.951) | -1.17 (0.774) | 0.31 (0.902) | 0.79 (0.799) | 0.92 (0.914) | 1.85 (0.85) |
| IC72 | 0.10 (0.951) | -0.53 (0.854) | 0.11 (0.955) | -0.48 (0.799) | 0.06 (0.986) | 0.02 (0.986) |
| IC73 | -0.53 (0.911) | -1.55 (0.748) | -0.31 (0.902) | -0.89 (0.799) | -0.80 (0.914) | 0.02 (0.986) |
| IC74 | -1.72 (0.664) | -2.09 (0.425) | -0.85 (0.884) | 0.51 (0.799) | -0.64 (0.914) | 0.51 (0.965) |
| IC75 | -0.93 (0.781) | 0.19 (0.961) | -0.75 (0.896) | 0.78 (0.799) | -0.92 (0.914) | 0.14 (0.978) |
| IC76 | 0.45 (0.914) | -1.29 (0.774) | -1.63 (0.794) | -0.90 (0.799) | -0.69 (0.914) | 0.32 (0.965) |
| IC77 | -1.18 (0.664) | -0.74 (0.854) | -1.12 (0.863) | 0.49 (0.799) | -1.67 (0.754) | 0.47 (0.965) |
| IC78 | -0.64 (0.839) | -1.20 (0.774) | -0.95 (0.863) | -1.09 (0.786) | -0.62 (0.914) | -0.48 (0.965) |
| IC79 | -0.95 (0.781) | -1.00 (0.854) | -0.16 (0.941) | -0.60 (0.799) | 0.48 (0.937) | -0.60 (0.965) |

**Supplemental Figures**


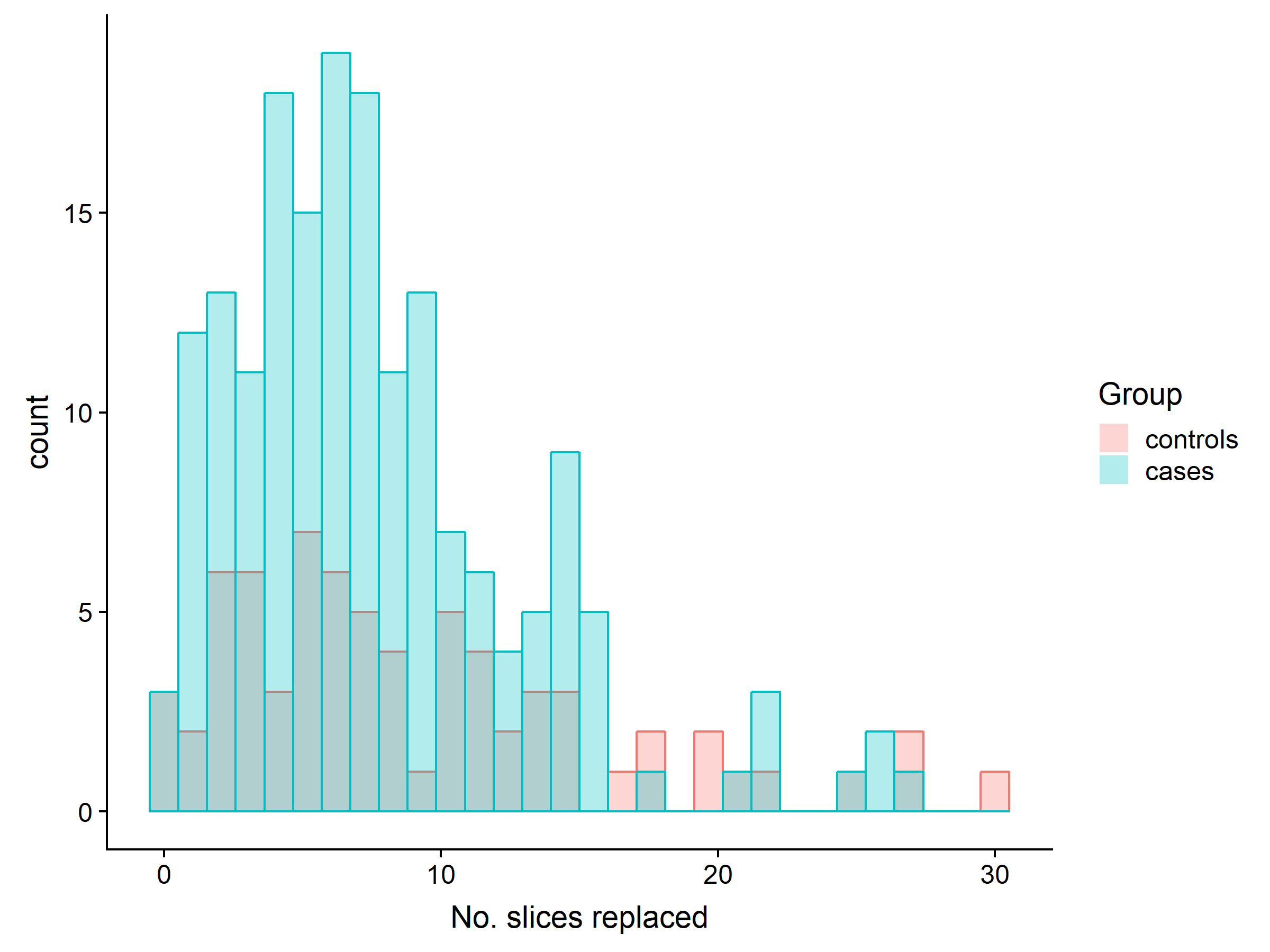


**Fig. S1.** The number of slices replaced in the diffusion weighted imaging data in eddy (based on non-parametric prediction by the Gaussian process). Importantly, the same slice can be replaced across diffusion weighted volumes.


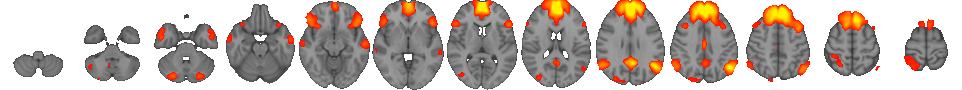


**Fig. S2.** DMN resting-state network extracted from the rs-fMRI data using FSL MELODIC


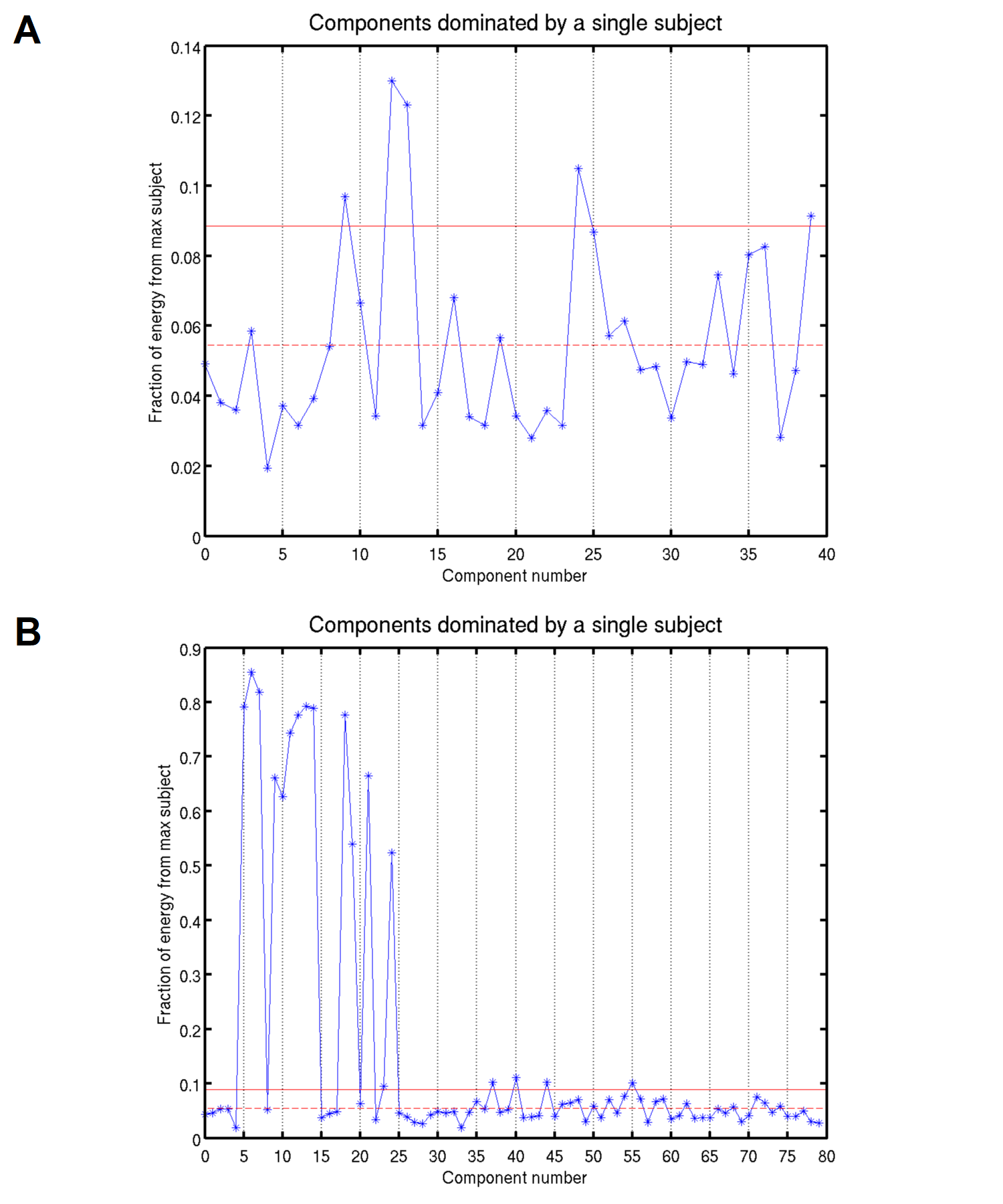


**Fig. S3**. Lineplots showing the degree to which a component is dominated by a single subject based on the fraction of energy in the decomposition with (A) 40 components (main analyses) and (B) 80 components (supplementary analyses). We discarded components with a subject dominance above 20%, i.e. 0 for the main decomposition (A) and13 for the higher order decomposition (B)


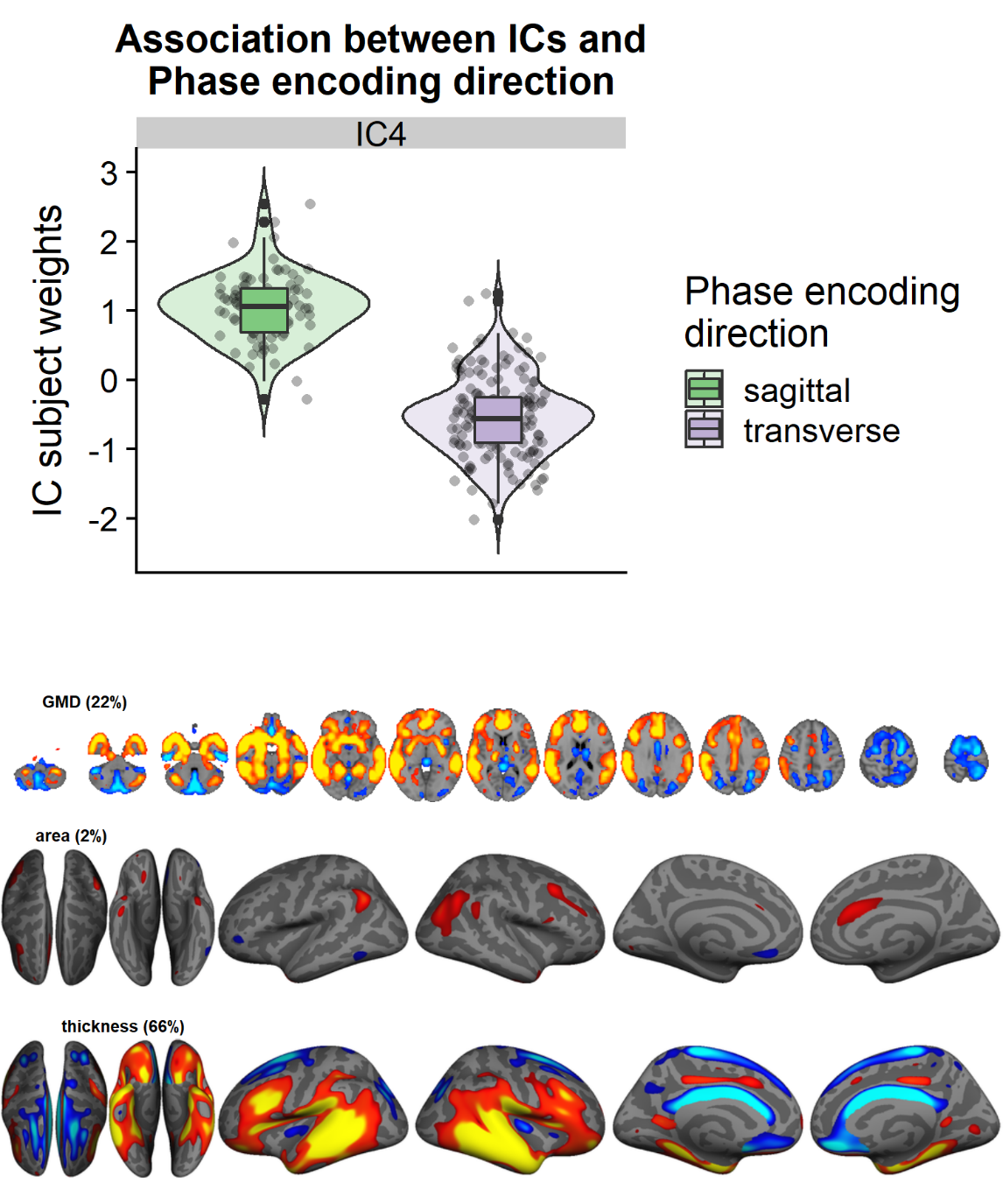


**Fig. S4.** The LICA component that was very sensitive to phase encoding direction (see top panel). The subject weights have been residualized for group, age and sex.


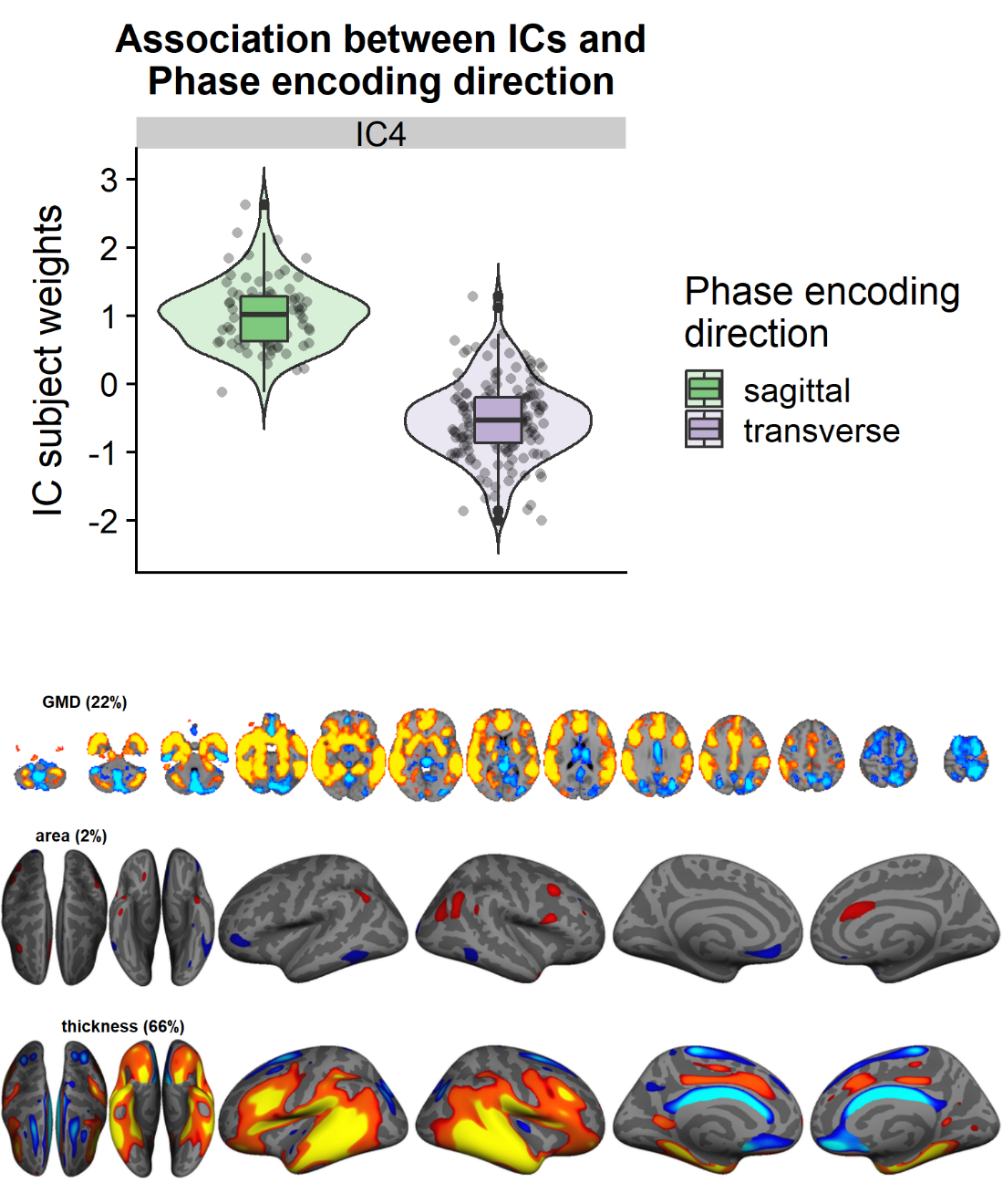


**Fig. S5.** The LICA component that was very sensitive to phase encoding direction (see top panel) in the higher decomposition. The subject weights have been residualized for group, age and sex.


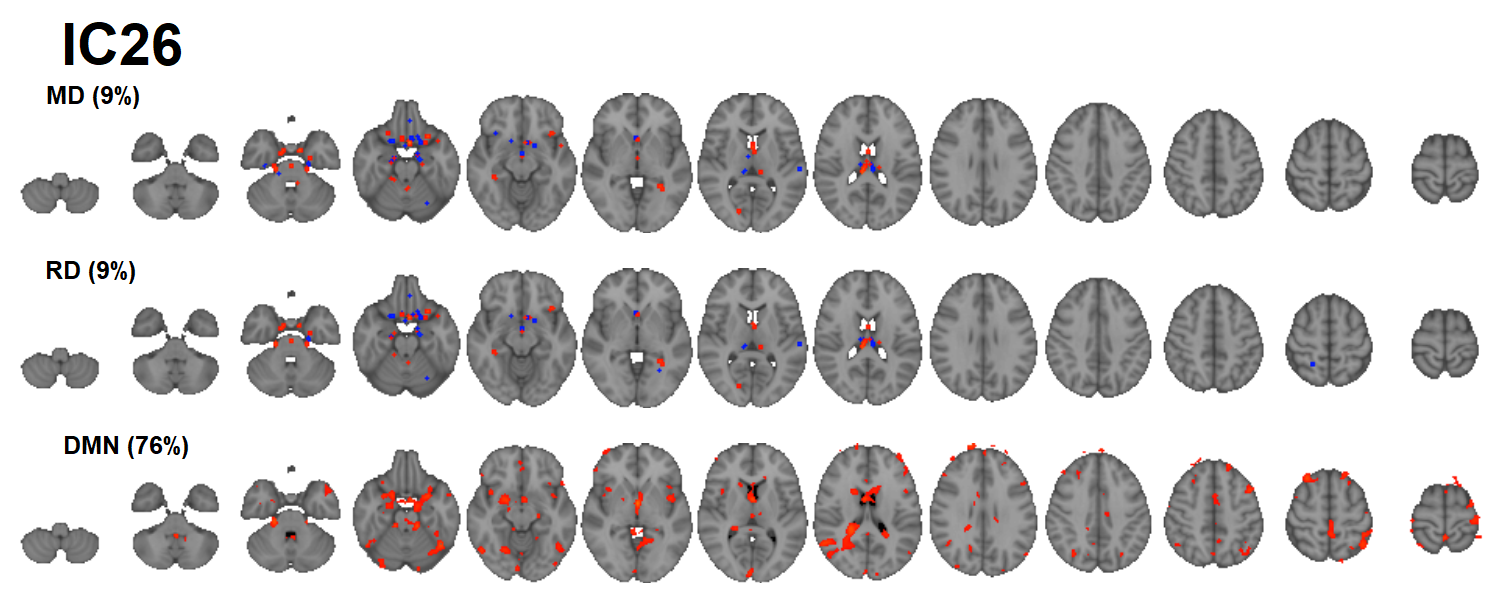


**Fig. S6.** The other component that had substantial contribution from resting-state DMN FC. Note that there is some degree of fusion with diffusion weighted measures.


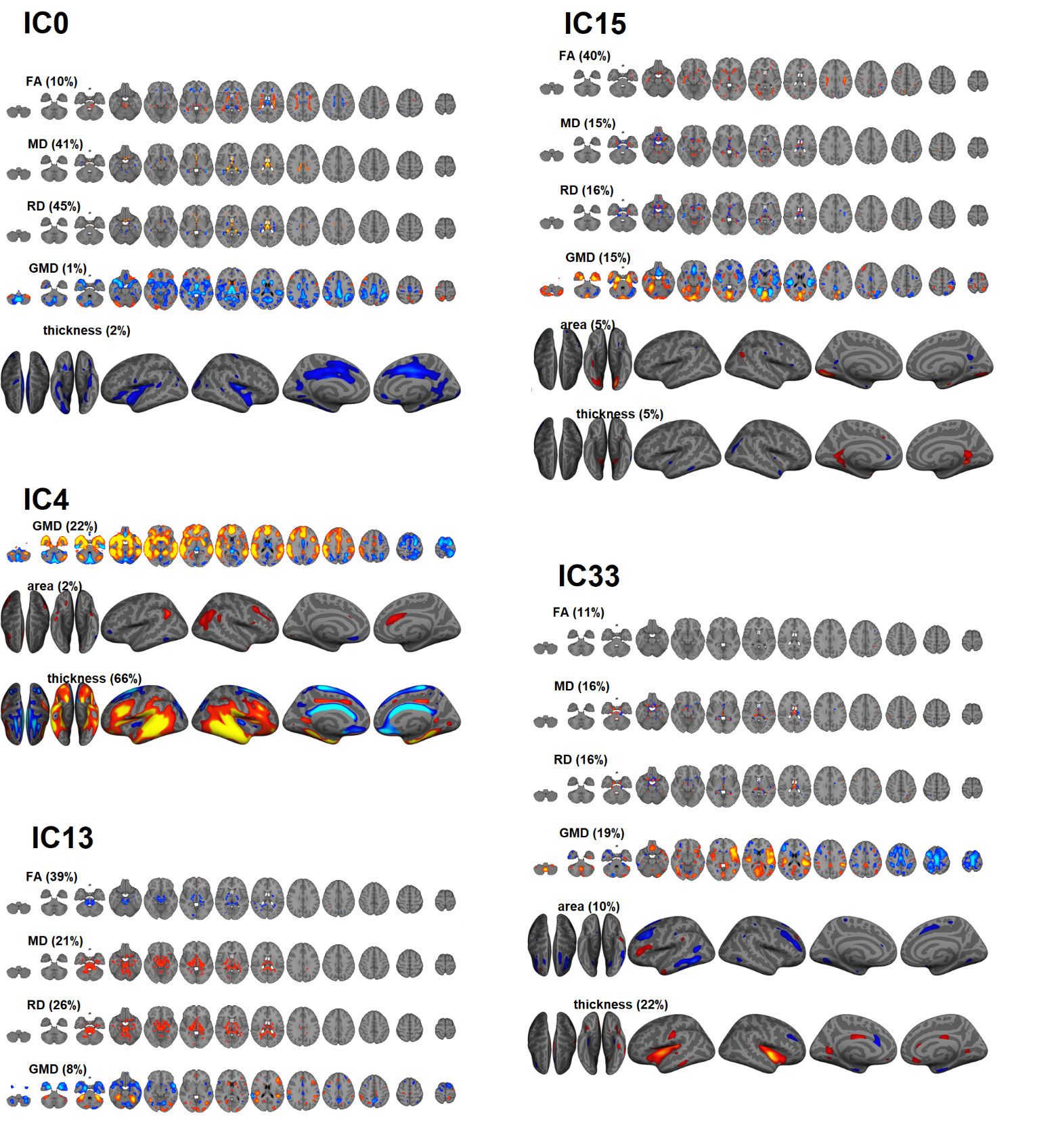


**Fig S7.** ICs characterized by region specific features that are associated with age or sex (FDR corrected p < 0.01). The specific associations are as follows: IC0 (age), IC4 (age), IC13 (age and sex), IC15 (sex), and IC33 (age).


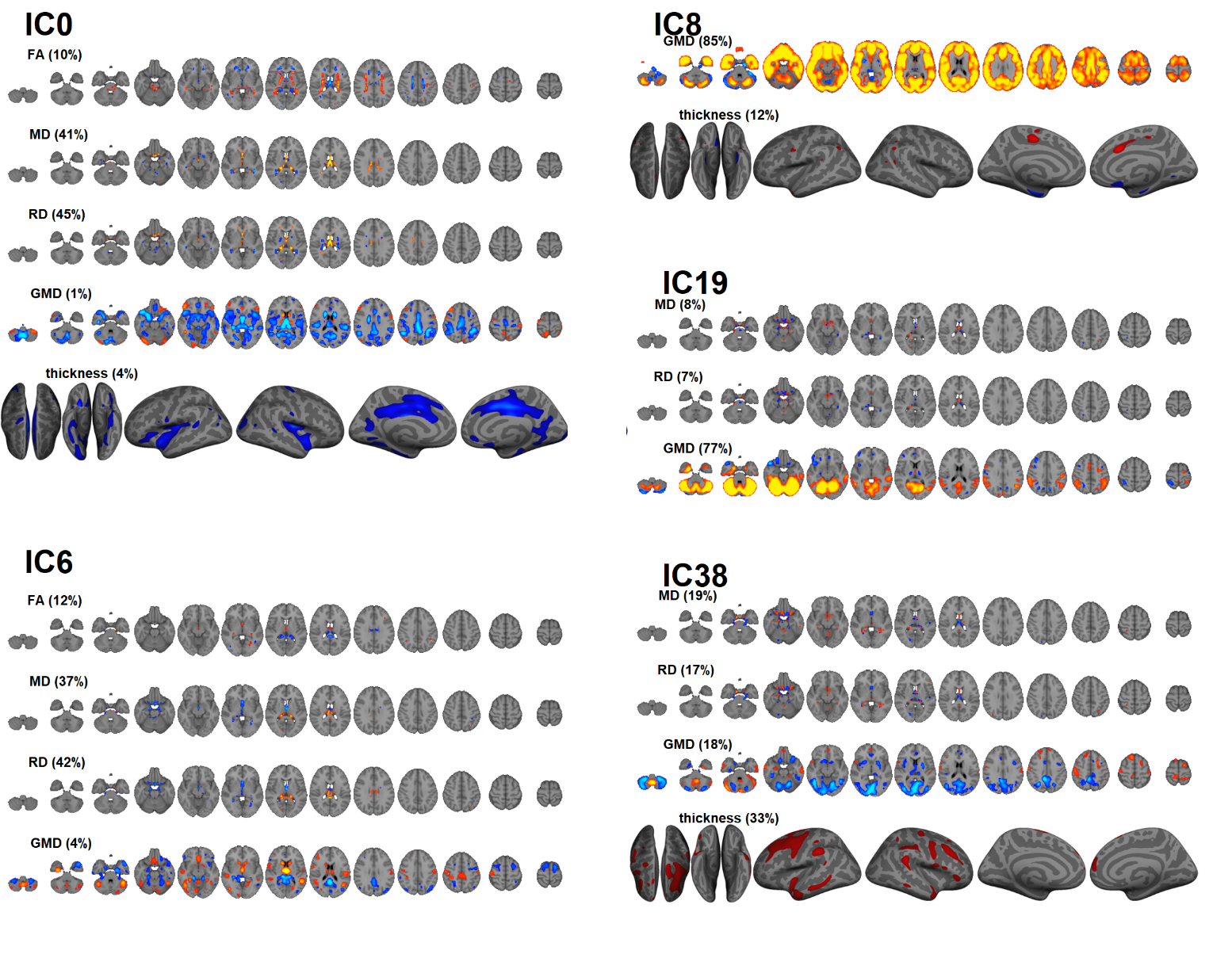


**Fig. S8.** Some of the top features (ICs) in the machine learning analyses. IC0 is among the top features for classifying case-control status and predicting symptom load for depression. IC6 is among the top features for predicting symptom loads for depression and anxiety. IC8 is among the top features for classifying case-control status and predicting symptom load for anxiety. IC19 is the top feature for classifying case-control status. IC38 is among the top features for predicting symptom loads for depression and anxiety.


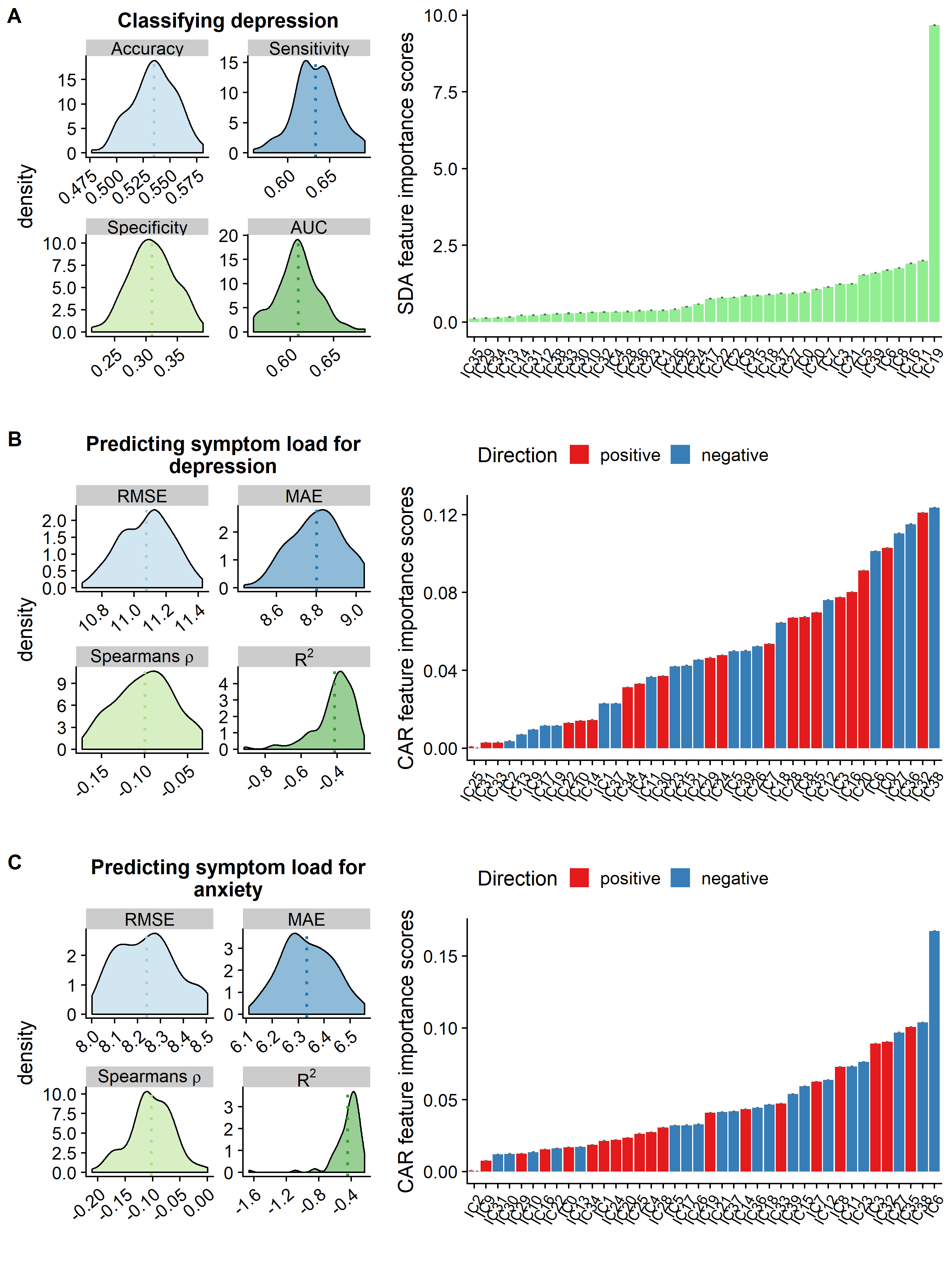


**Fig. S9.** The results of the supplementary analyses of the machine learning approach using 10-fold cross-validation with 100 repetitions for (A) classifying case-control status (B) prediction symptom load for depression and (C) symptom load for anxiety. Here, age, sex and phase encoding were regressed out from the subject weights of all the ICs. The figures on the left show prediction accuracy based on various model performance metrics, with the dotted lines denoting the mean. The barplots on the right show the most important features for each model based on CAT-scores (A) or CAR-scores (B and C). Direction shows whether the feature is positive or negatively associated with a given model.


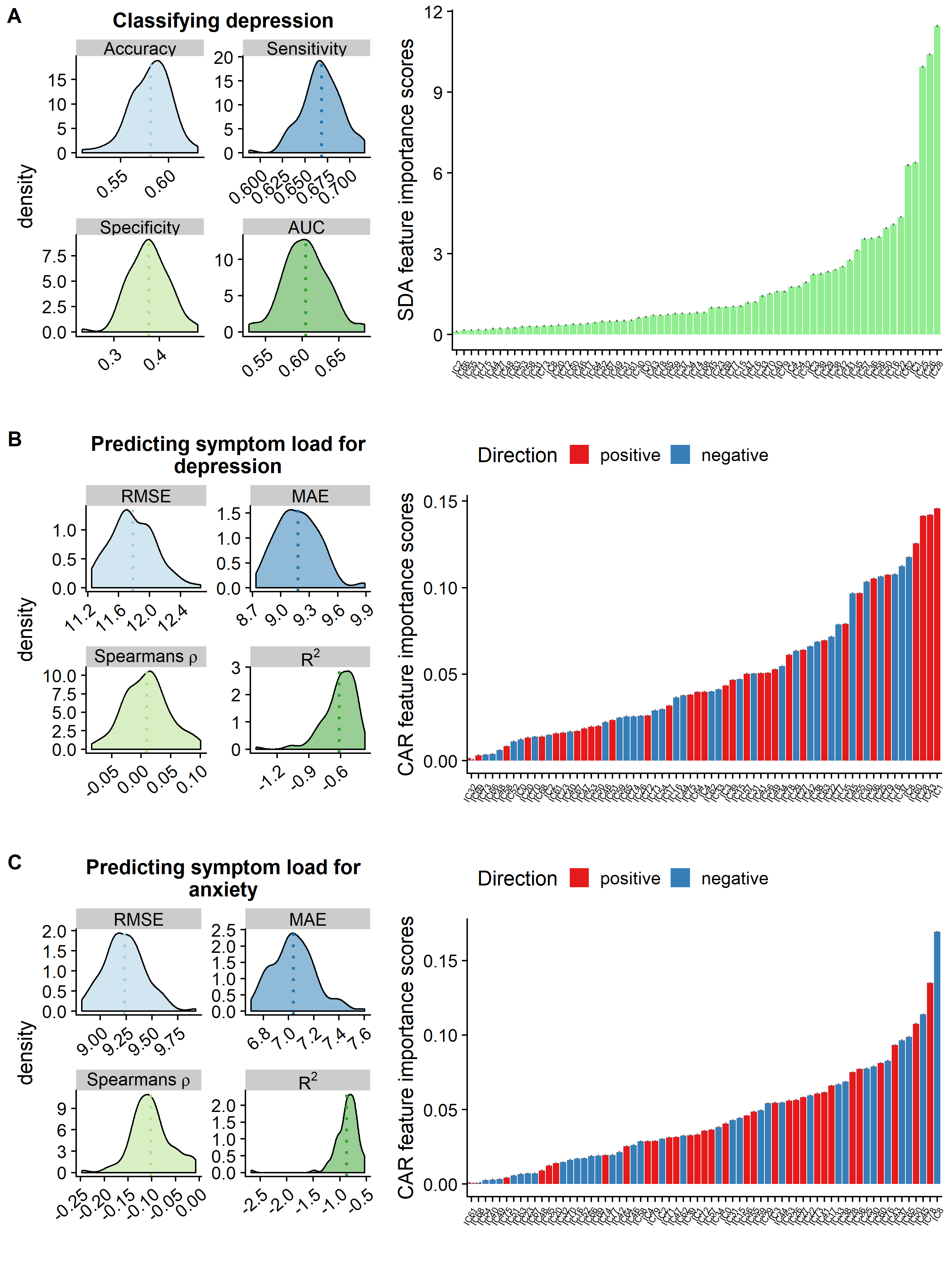


**Fig. S10.** The results of the machine learning approach based on the 80 IC decomposition (67 ICs in total) using 10-fold cross-validation with 100 repetitions for (A) classifying case-control status (B) prediction symptom load for depression and (C) symptom load for anxiety. Here, phase encoding direction was only regressed out of the subject weights in IC4, while age and sex were regressed out from the subject weights of all the ICs. The figures on the left show prediction accuracy based on various model performance metrics, , with the dotted lines denoting the mean. The barplots on the right show the feature importance of each IC for each model based on CAT-scores (A) or CAR-scores (B and C). Direction shows whether the feature is positive or negatively associated with a given model.


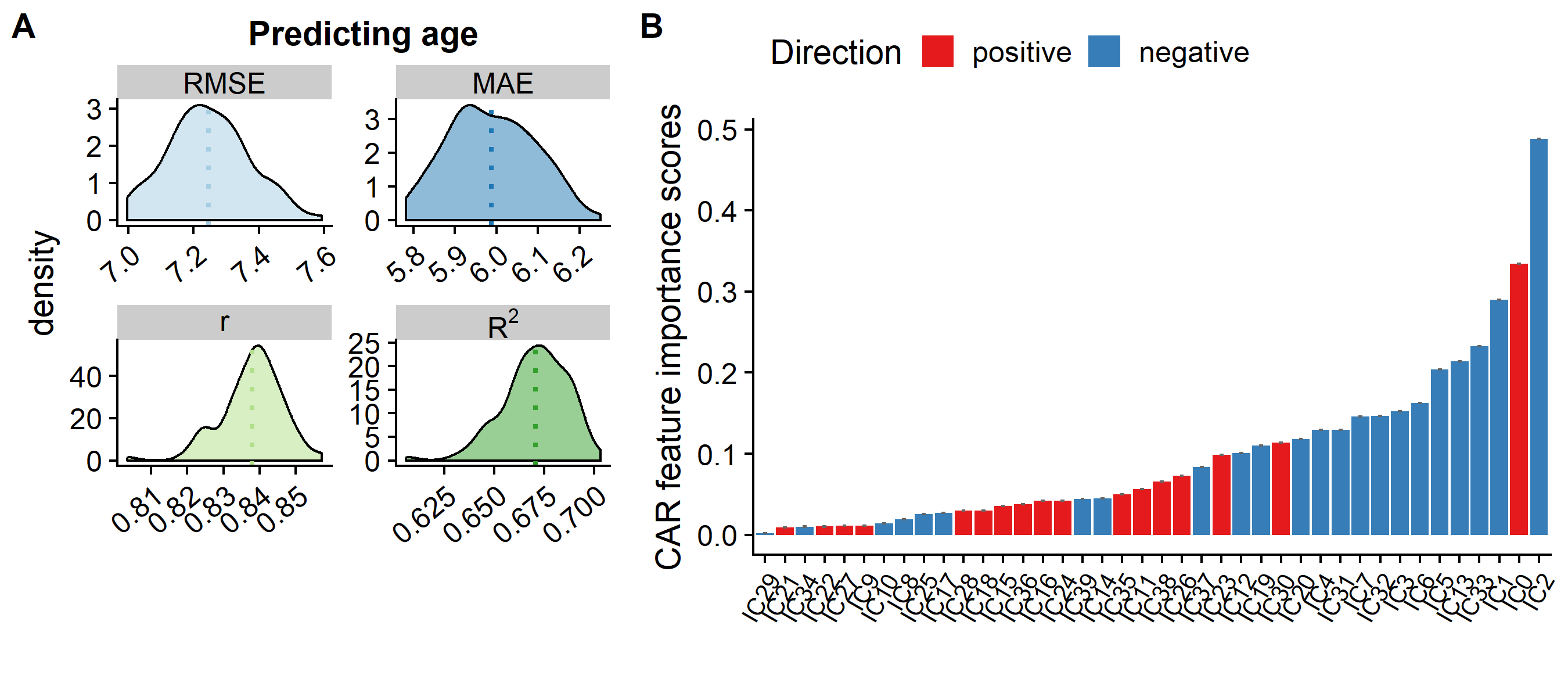


**Fig. S11.** Age prediction based on machine learning using 10-fold cross-validation across 100 repetitions. Here, phase encoding direction is regressed out from the subject weights of all the ICs. (A) model performance results, with the dotted lines denoting the mean. (B) feature importance based on CAR-scores. Direction shows whether the features is positive or negatively associated with age.


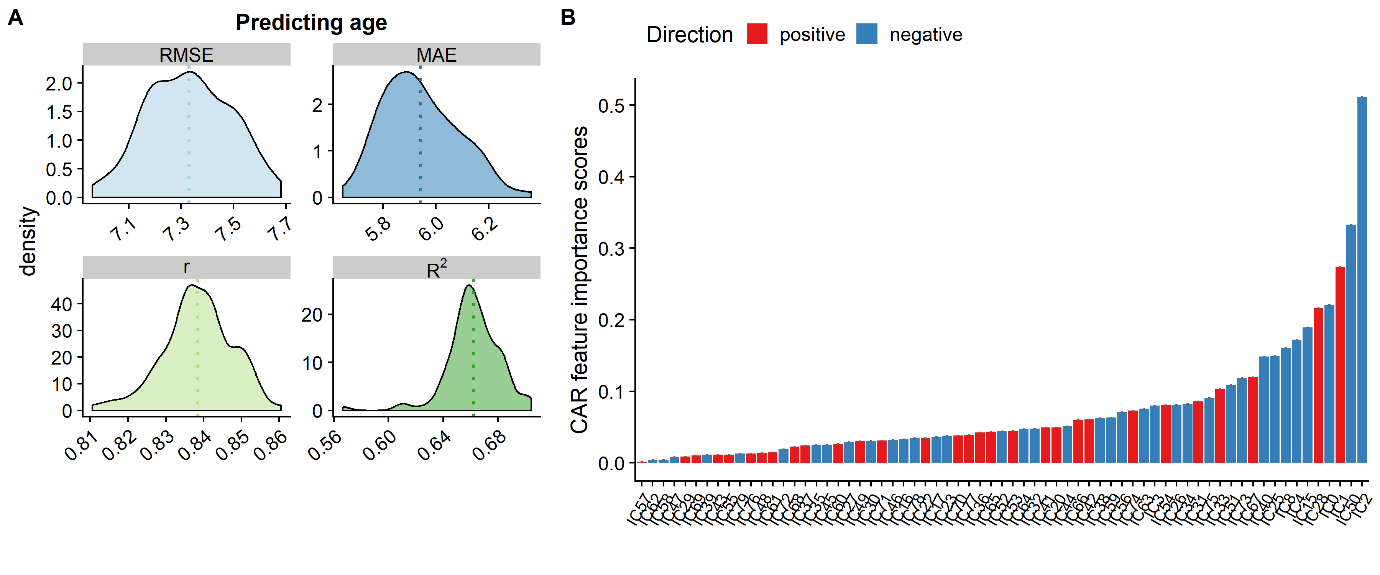


**Fig. S12**. Age prediction based on the higher order decomposition (67 ICs in total) using 10-fold cross-validation across 100 repetitions. Here, phase encoding direction is regressed out from the subject weights of IC4. (A) model performance results, with the dotted lines denoting the mean. (B) feature importance based on CAR-scores. Direction shows whether the features is positive or negatively associated with age.


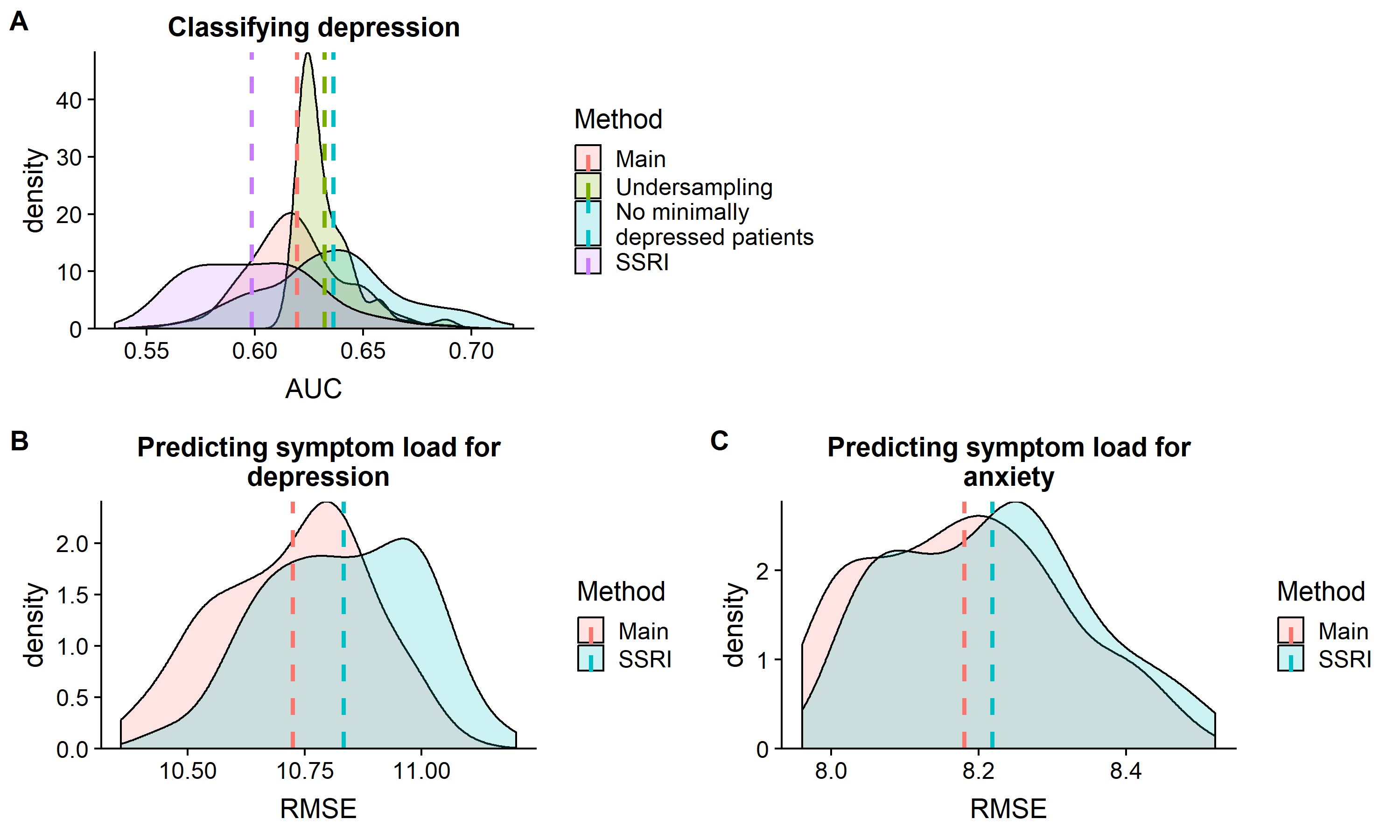


**Fig S13.** Comparing cross-validated results that have and have not been accounted for various confounders, based on the LICA decomposition with 40 ICs for (A) group classification, (B) predicting depression symptom load and (C) predicting anxiety symptom load. See the Supplemental Methods for full details. Main: pertains to the results of the main analyses. Undersampling: accounting for imbalance in cases and controls by random undersampling (i.e. such that the groups consist of equal amounts of cases and controls). No minimally depressed patients: excluding patients that had minimal depression based on BDI-II sum score criteria, and controls with moderate depression. SSRI: regressing out SSRI medication use from the subject weights of the ICs.
